# Supplementary material for: Cancer tracking system improves timeliness of liver cancer care at a Veterans Hospital: A comparison of cohorts before and after implementation of an automated care coordination tool
Source: PLOS Digit Health. 2022 Aug 1;1(8):e0000080. doi: 10.1371/journal.pdig.0000080 (PMC9931271; doi:10.1371/journal.pdig.0000080)
Supplement: Attachment — Submitted filename: PLOS Digital Health Response to Reviewers 5.3.22.pdf [file pdig.0000080.s001.pdf]

Yale SCHOOL OF MEDICINE
Department of Internal Medicine

Section of Digestive Diseases

PO Box 208019
New Haven CT 06520-8019
T 203 785-7312
F 203 785-7273
medicine.yale.edu/intmed/digestivediseases

location
Laboratory for Medicine and Pediatrics (LMP)
Room 1080
15 York Street
New Haven CT 06510

courier
The Anlyan Center (TAC)
Room S241
One Gilbert Street
New Haven CT 06519

May 3, 2022
Professor Shlomo Berkovsky, PhD
Section Editor: Human Computer Interface
PLOS Digital Health

Dear Professor Berkovsky,

Thank you very much for your review of our manuscript titled "Cancer tracking system improves timeliness of liver cancer care at a Veterans Hospital: a comparison of cohorts before and after implementation of an automated care coordination tool". We appreciate the questions raised by Dr. David Bentrem (Reviewer # 1) and believe that our manuscript has been improved by adding the requested clarifications. Please find our responses below.

Question 1. Was the tumor board up and running in the pre implementation period?
<u>Author response</u>: Thank you for this question and the opportunity to clarify the setting of our intervention. There was a multidisciplinary (multicancer) gastroenterology and surgery tumor board in place prior to the study period. The liver tumor board began in February, 2009 and was not in place for the pre-intervention period. This liver tumor board was operational for 23 months prior to measurement of the tracking system intervention. This has been clarified in the introduction and methods sections of the manuscript.

Question 2. Please discuss why the time to see a specialist was not different between groups but the time from diagnosis to treatment was less?
<u>Author response</u>: Thank you for this interesting question. Localized tumors in patients with adequate hepatic functional reserve (BCLC stage 0 through B) can be treated with embolization or ablation by Interventional Radiology. At the time of diagnosis and staging, appropriate patients may be referred directly for these procedural treatments. A greater proportion of patients were diagnosed with early stage disease in the post-intervention period. Stratifying by BCLC stage at diagnosis, we found that the decrease in time from diagnosis to first treatment was only significantly shorter only in patients with BCLC stage A disease (Table 3). Improvements in timely referral for embolization or ablation of early-stage HCC appear to drive the observed decrease in time to first treatment even where time to first visit with a hepatologist, oncologist, or surgeon was not significantly shorter. We have added this interpretation to the discussion section of the manuscript.

Again, thank you for your review and clarifying questions. We hope that we have been able to provide sufficienct clarification for publication in *PLOS Digital Health*. Should you have any additional questions or concerns, please do not hesitate to contact me.

Sincerely,

Tamar H. Taddei, MD
Professor of Medicine, Yale School of Medicine
Director, Liver Cancer Program, VA Connecticut Healthcare System
Email: tamar.taddei@yale.edu; tamar.taddei@va.gov | Phone: 203-932-5711 ext. 4412
